# Associations of Depression and Antidepressant Use with the Risk of Tuberculosis Sequelae: Insight from Genetic Analysis

**DOI:** 10.3390/genes17101198

**Published:** 2026-09-28

**Authors:** Tong Chen, Liya Ge, Dongliang Zhang, Yinlu Zhang, Haifeng Zhang, Yinan Wang

**Affiliations:** 1 Department of Tuberculosis Control and Prevention, Ningbo Municipal Center for Disease Control and Prevention, Ningbo 315010, China; dylan108108@sina.com (T.C.);; 2 Wuhan Municipal Center for Disease Control and Prevention, Wuhan 430021, China; geliya0530@sina.com; 3 Department of Tuberculosis Control and Prevention, Beilun District Center for Disease Control and Prevention, Ningbo 315899, China; 4 Department of Nutrition and Food Hygiene, Hubei Key Laboratory of Food Nutrition and Safety, MOE Key Laboratory of Environment and Health, School of Public Health, Tongji Medical College, Huazhong University of Science and Technology, Wuhan 430030, China

**Keywords:** depression, antidepressant, tuberculosis, sequelae, Mendelian randomization

## Abstract

**Background**: Depression and antidepressant use have been suggested to be associated with tuberculosis sequelae, but whether these associations are causal remains unclear. We aimed to investigate the potential associations of genetically predicted depression and antidepressant use with the risk of tuberculosis sequelae using a two-sample Mendelian randomization (MR) study. **Methods**: Summary statistics for depression (13,559 cases and 435,855 controls), antidepressant use (33,757 cases and 270,405 controls), and tuberculosis sequelae (727 cases and 497,160 controls) were extracted from large-scale genome-wide association studies of European-ancestry participants. MR estimates were generated using the inverse-variance weighted (IVW) method in the main analysis with a series of sensitivity analyses. **Results**: In the primary fixed-effects IVW analysis, genetically predicted depression showed suggestive evidence of an association with an increased risk of tuberculosis sequelae (OR = 1.218, 95% CI: 1.003–1.480, *p* = 0.047), whereas genetically predicted antidepressant use showed suggestive evidence of an inverse association with the risk of tuberculosis sequelae (OR = 0.692, 95% CI: 0.497–0.964, *p* = 0.029). Both primary estimates provided suggestive evidence of associations, although they did not meet the Bonferroni-corrected significance threshold of *p* < 0.025. In the multiplicative random-effects IVW sensitivity analyses, the effect directions remained unchanged although statistical significance was not retained, while other complementary sensitivity analyses generally yielded directionally consistent estimates. **Conclusions**: Our findings provide novel, hypothesis-generating genetic evidence that genetically predicted depression and antidepressant use may be associated with the risk of tuberculosis sequelae. These findings highlight potentially important links between mental health-related factors and long-term tuberculosis outcomes and warrant further validation in independent studies.

## 1. Introduction

Tuberculosis, a chronic infectious disease caused by *Mycobacterium tuberculosis* (Mtb), remains the world’s leading cause of death from a single infectious agent and ranks among the top ten causes of mortality globally [1,2]. In 2024, tuberculosis resulted in approximately 1.23 million deaths worldwide, with a mortality rate of 15.1 per 100,000 population [3]. Even after microbiological cure, tuberculosis patients continue to suffer from persistent sequelae, such as respiratory, neurological, and musculoskeletal impairments, which impose a significant long-term burden [4,5,6]. It was estimated that tuberculosis sequelae contributed to up to half of all tuberculosis-related disability-adjusted life years globally [7]. Therefore, it is essential to identify modifiable prognostic markers that could guide risk stratification and personalized interventions to improve long-term outcomes for tuberculosis patients.

Depression, a highly prevalent mental health disorder, is common among patients with tuberculosis, with prevalence estimates ranging from 40% to 70% [8,9]. Observational studies have consistently linked depression to a range of adverse tuberculosis outcomes, including higher rates of treatment failure as well as increased disability, mortality, and reduced quality of life [10,11,12,13]. While antidepressant therapy is the cornerstone of depression management, direct population-level evidence on its specific impact on the long-term risk of tuberculosis sequelae remains limited. Preclinical studies suggested that certain antidepressants may possess immunomodulatory or even direct antimicrobial properties relevant to Mtb infection [14,15]. However, conventional observational designs are vulnerable to residual confounding and reverse causation, so it was still unclear whether the observed associations were causal or attributable to shared underlying factors. Therefore, clarifying the causal roles of both depression and antidepressant use is of clinical and public health importance for developing targeted interventions to improve long-term tuberculosis outcomes.

Mendelian randomization (MR) is a widely used method exploiting genetic variants associated with exposure as instrumental variables (IVs) to estimate the potential causal association between the exposure and the outcome [16]. Since these genetic variants are randomly allocated during conception, and genetic variants are relatively independent of environmental factors and established before the onset of disease, MR studies could largely avoid residual confounding and reverse causation inherent to observational studies [17,18]. To date, no MR study has specifically investigated the causal effects of either depression or antidepressant use on the risk of tuberculosis sequelae. Fortunately, large-scale genome-wide association studies (GWASs) for depression, antidepressant use, and tuberculosis-related phenotypes are now available, enabling a robust two-sample MR design [19,20]. Herein, we conducted a comprehensive two-sample MR analysis to estimate the potential causal associations of genetically predicted depression and antidepressant use with the risk of tuberculosis sequelae.

## 2. Methods

### 2.1. Study Design

We conducted a two-sample MR study to evaluate the causal association of depression and antidepressant use with the risk of tuberculosis sequelae. There are three core assumptions for MR design: (1) IVs are strongly associated with the exposure; (2) IVs are not associated with any confounders related to exposure and outcome; and (3) IVs affect outcomes only through the effects on exposures (Figure 1). The summary-level data used in the present study were derived from publicly available European-descent GWASs, and the detailed information on data sources is shown in Table 1 [19,20]. The protocol and data collection were approved by the ethics committee of the original GWASs, and written informed consent was obtained from each participant before data collection.

### 2.2. Data Sources

Datasets were selected based on the availability of genome-wide summary statistics, European ancestry, concordance of phenotype definitions with the prespecified exposures and outcome, and suitability for MR analysis.

Summary statistics for depression and antidepressant use were obtained from GWASs of European-ancestry participants [19]. The depression GWAS was derived from a meta-analysis of UK Biobank and FinnGen release 3 and included 449,414 participants (13,559 cases and 435,855 controls), whereas the antidepressant-use GWAS was based exclusively on UK Biobank data and included 304,162 participants (33,757 cases and 270,405 controls). In UK Biobank, depression cases were identified using phecode 296.22 (major depressive disorder), derived from ICD-coded diagnoses, with controls defined according to the corresponding phecode-based case–control criteria. In FinnGen, depression cases were identified using the F5_DEPRESSIO endpoint, which included depressive episode (ICD-10 F32) and recurrent depressive disorder (ICD-10 F33) recorded in hospital discharge or cause-of-death registries, whereas individuals with mood disorders were excluded from the control group. Accordingly, the depression exposure represented a clinically coded depression phenotype rather than recurrent depressive disorder alone. Antidepressant use was defined according to ATC code N06A and represented a broad phenotype of any antidepressant use, without distinction by specific drug class, dose, or treatment duration.

Summary statistics for tuberculosis sequelae were obtained from FinnGen release 12, including 727 cases and 497,160 controls of European ancestry [20]. The outcome was defined using the predefined FinnGen endpoint AB1_SEQULAE_TUBERCU, based on hospital discharge and cause-of-death records coded according to ICD-10 B90. This composite endpoint included sequelae of central nervous system tuberculosis (B90.0), genitourinary tuberculosis (B90.1), tuberculosis of bones and joints (B90.2), tuberculosis of other organs (B90.8), and respiratory or unspecified tuberculosis (B90.9). Controls were defined according to the FinnGen endpoint rules and excluded individuals classified under the broader endpoint of sequelae of infectious and parasitic diseases. Accordingly, individuals with a history of tuberculosis but without a recorded B90 sequela diagnosis could have been included in the control group. This predefined composite phenotype was retained to ensure consistency with the publicly available FinnGen GWAS summary statistics.

### 2.3. Genetic Instruments for Exposures

At the conventional genome-wide significance threshold (*p* < 5 × 10^−8^), no eligible independent single nucleotide polymorphisms (SNPs) were available for depression and only one was available for antidepressant use after instrument selection and harmonization, precluding a meaningful multi-variant MR analysis. Therefore, a more liberal threshold of *p* < 5 × 10^−6^ was adopted to obtain a sufficient number of instruments for exploratory MR analyses. Specifically, SNPs associated with the exposures at *p* < 5 × 10^−6^ were selected and clumped for linkage disequilibrium (LD) using an *r*^2^ threshold of <0.001 within a 10,000-kb window based on the European reference panel of the 1000 Genomes Project. When SNPs were in LD, the variant with the lowest *p* value for the exposure association was retained. A total of 148 and 381 SNPs met the initial significance threshold for depression and antidepressant use, respectively, of which 22 and 32 independent SNPs remained after LD clumping. All retained SNPs were directly available in the FinnGen outcome dataset, so no proxy variants were used. Exposure and outcome alleles were harmonized using the harmonise_data function in TwoSampleMR (action = 2), with allele-frequency information used to resolve palindromic variants. Palindromic variants with ambiguous allele frequencies (minor allele frequency >0.42) were excluded. One palindromic SNP for depression and eight for antidepressant use were identified, all of which were successfully harmonized and retained, resulting in 22 and 32 final instruments, respectively (Figure S1). In addition, instrument strength was assessed using the F-statistic, with F-statistic > 10 considered to indicate adequate instrument strength, although this threshold does not exclude other sources of instrument-related bias or pleiotropy [21].

### 2.4. Statistical Analysis

In the primary analysis, the inverse-variance weighted (IVW) method [22] was employed to estimate the association of depression and antidepressant use with the risk of tuberculosis sequelae. Heterogeneity across IVs was evaluated using Cochran’s Q statistic [23]. The choice of the primary IVW model was based on the heterogeneity assessment. Specifically, a fixed-effects IVW model was applied when no statistically significant heterogeneity was detected, whereas a multiplicative random-effects IVW model was used when significant heterogeneity was present. The alternative IVW model was additionally used as a sensitivity analysis. Additionally, minimum detectable effect sizes were estimated for the primary IVW analyses based on the precision of the corresponding IVW estimates, assuming 80% statistical power and a two-sided α level of 0.05, following the general framework for statistical power assessment in MR studies [24].

Given that MR estimates derived from the IVW method might be susceptible to biases from invalid IVs or horizontal pleiotropy [25], we performed a series of sensitivity analyses using complementary approaches to evaluate the robustness of the associations identified by the IVW method in our primary analysis. First, the maximum likelihood method was applied to obtain more reliable causal estimates in the presence of measurement error in the SNP-exposure effect [26]. Second, the weighted median [23] and weighted mode [27] methods were used to assess the robustness of the estimates under alternative assumptions regarding invalid instruments. Third, the MR pleiotropy residual sum and outlier (MR-PRESSO) method was employed to detect potential outliers and provide robust MR estimates after outlier correction [28]. Fourth, the MR robust adjusted profile score (MR-RAPS) method, which can account for systematic and idiosyncratic pleiotropy, was implemented to obtain robust estimates in analyses involving many weak or pleiotropic IVs [29]. Fifth, the constrained maximum likelihood and model averaging (cML-MA) method was performed to make a reliable inference despite the existence of invalid IVs with correlated or uncorrelated pleiotropic effects [30]. Sixth, we utilized the generalized summary data-based MR (GSMR) method to accurately make a causal inference after excluding SNPs with pleiotropy that were identified by the heterogeneity in dependent instruments outlier analysis [31]. Seventh, we conducted the MR-PRESSO global test to detect significant overall pleiotropic distortion across IVs [28], and additionally estimated the MR-Egger intercept to quantify potential directional pleiotropy [32]. Eighth, leave-one-out analyses were performed to evaluate whether the results were disproportionately influenced by individual SNPs, and funnel plots were visually inspected for asymmetry in single-SNP estimates [26]. Finally, to assess the plausibility of the hypothesized causal direction, the Steiger directionality test was adopted by comparing the total phenotypic variance explained by the genetic instruments for the exposure versus the outcome. Evidence was considered supportive of the hypothesized exposure-to-outcome direction when the genetic instruments explained a significantly greater proportion of phenotypic variance in the exposure than in the outcome and the *p_Steiger_* value reached the significance threshold [33].

We additionally performed multivariable MR including depression and antidepressant use simultaneously to evaluate their mutually adjusted associations with tuberculosis sequelae. To maintain consistency with the primary univariable MR analyses, candidate variants associated with either exposure at *p* < 5 × 10^−6^ were combined and jointly clumped using an *r^2^* threshold of <0.001 within a 10,000-kb window based on the European reference panel of the 1000 Genomes Project. Genetic associations of the retained variants with both exposures and tuberculosis sequelae were extracted and harmonized by aligning effect alleles using the same harmonization procedure as in the univariable analyses. Multivariable IVW analysis was then performed. Conditional instrument strength was assessed using conditional F-statistics. Potential heterogeneity and horizontal pleiotropy in the multivariable MR analysis were further evaluated using the modified Cochran’s Q statistic and the multivariable MR-Egger intercept, respectively.

To further assess potential horizontal pleiotropy, a phenome-wide association study (PheWAS) was performed for all instrumental SNPs using the phewas function implemented in the ieugwasr R package and publicly available GWAS data from the IEU OpenGWAS database [26,34]. The PheWAS search was conducted on 10 September 2026, and genome-wide significant secondary associations were retrieved at a threshold of *p* < 5 × 10^−8^. The returned phenotypes were subsequently screened in a case-insensitive manner using predefined keywords related to smoking, socioeconomic factors, healthcare use, psychiatric comorbidity, and inflammation-related traits. Instrumental variants showing potentially relevant secondary associations were then excluded in an additional sensitivity analysis using the fixed-effects IVW method.

Because the depression GWAS included participants from FinnGen release 3 and the tuberculosis sequelae GWAS was derived from FinnGen release 12, potential sample overlap could not be excluded. In contrast, no evident sample overlap was expected between the antidepressant-use GWAS from UK Biobank and the FinnGen outcome dataset. However, because only summary-level data were available, the exact number and proportion of overlapping participants could not be determined and were therefore considered unknown. To further assess potential sample overlap, we calculated the cross-trait intercept and corresponding standard error using bivariate LD score regression (LDSC), with an intercept within one standard error of zero indicating no or negligible overlap-related error covariance [35]. Furthermore, MRlap analysis (https://github.com/n-mounier/MRlap, accessed on 28 February 2026) was performed to evaluate the potential influence of sample overlap on the IVW-MR estimates while additionally accounting for weak-instrument bias and winner’s curse [36].

All MR results are presented as odds ratios (ORs) with corresponding 95% confidence intervals (CIs). Given the two prespecified primary exposures, a Bonferroni-corrected threshold of *p* < 0.025 (0.05/2) was additionally considered for multiple testing. Associations with *p* values between 0.025 and 0.05 were considered suggestive evidence. Accordingly, the analyses were hypothesis-driven, and findings not meeting the Bonferroni-corrected threshold were interpreted as suggestive rather than confirmatory. All analyses were performed using the statistical program R (version 4.1.1; The R Foundation for Statistical Computing) with R packages TwoSampleMR (version 0.6.22), MRPRESSO (version 1.0), MRcML (version 0.0.0.9000), gsmr (version 1.1.0), MVMR (version 0.3), MendelianRandomization (0.7.0), ieugwasr (version 1.1.0), MRlap (version 0.0.3.3), forestplot (version 3.1.7), and ggplot2 (version 4.0.0).

## 3. Results

### 3.1. Characteristics of Genetic Instruments

Finally, a total of 22 and 32 independent SNPs were selected as IVs for depression and antidepressant use, respectively. The phenotypic variance explained by these SNPs was approximately 0.11% for depression and 0.25% for antidepressant use. The mean F-statistic was 22.38 (range: 21.00–26.67) for depression and 23.39 (range: 20.74–29.98) for antidepressant use, with all F-statistics exceeding 10, indicating that the instruments satisfied the conventional F-statistic criterion for instrument strength, although weak-instrument bias could not be completely excluded. Detailed characteristics of the included SNPs are provided in Tables S1 and S2.

### 3.2. Associations of Depression and Antidepressant Use with the Risk of Tuberculosis Sequelae

As shown in Table S3, Cochran’s Q test revealed no significant heterogeneity among the IVs (all *p* > 0.05). Accordingly, the fixed-effects IVW model was used as the primary analysis for both exposures, while the multiplicative random-effects IVW model was examined as a sensitivity analysis. The primary fixed-effects IVW analysis showed suggestive evidence that genetically predicted depression was associated with an increased risk of tuberculosis sequelae (OR = 1.218, 95% CI: 1.003–1.480, *p* = 0.047), whereas genetically predicted antidepressant use was inversely associated with the risk of tuberculosis sequelae (OR = 0.692, 95% CI: 0.497–0.964, *p* = 0.029) (Figure 2). Both primary estimates provided suggestive evidence of associations, although they did not meet the Bonferroni-corrected significance threshold of *p* < 0.025. In the multiplicative random-effects IVW sensitivity analyses, the effect directions were unchanged, although the associations did not reach conventional statistical significance. In addition, the IVW-derived associations of individual genetic instruments for depression and antidepressant use with the risk of tuberculosis sequelae are displayed in Figure 3. At 80% statistical power and a two-sided α level of 0.05, the minimum detectable ORs were 1.321 for depression and 1.604 for antidepressant use on the risk-increasing scale; the corresponding thresholds for inverse associations were 0.757 and 0.623, respectively.

### 3.3. Sensitivity Analysis

Sensitivity analyses using complementary approaches generally yielded effect estimates that were directionally consistent with the primary IVW findings (Figure 2). Notably, for genetically predicted depression, all methods produced ORs greater than 1 that were numerically close to the primary IVW estimate, indicating directional consistency despite borderline statistical significance. In contrast, for genetically predicted antidepressant use, nominally significant inverse associations were observed across multiple methods including maximum likelihood (OR = 0.683, 95% CI: 0.480–0.970, *p* = 0.033), MR-RAPS (OR = 0.655, 95% CI: 0.456–0.940, *p* = 0.022), cML-MA (OR = 0.673, 95% CI: 0.472–0.959, *p* = 0.028), and GSMR (OR = 0.693, 95% CI: 0.493–0.976, *p* = 0.036). Figure 3 visualizes the association of each genetic instrument for depression and antidepressant use with the risk of tuberculosis sequelae across all sensitivity analyses.

The MR-PRESSO global test and MR-Egger intercept provided no statistical evidence of overall or directional horizontal pleiotropy, respectively (all *p* > 0.05) (Table 2). In addition, leave-one-out analyses showed that sequential exclusion of individual SNPs did not materially alter the direction of the estimates for either depression or antidepressant use (Figure S2). Funnel plots did not reveal pronounced asymmetry in the distribution of single-SNP estimates (Figure S3).

The Steiger directionality test indicated a significantly greater proportion of phenotypic variance explained for the exposure versus the outcome for both depression (*p_Steiger_* = 1.78 × 10^−35^) and antidepressant use (*p_Steiger_* = 5.99 × 10^−68^), supporting the hypothesized exposure-to-outcome direction (Table S4).

### 3.4. Multivariable MR Analysis

After joint instrument selection and harmonization, 51 independent IVs were retained for the multivariable MR analysis. The conditional F-statistics were 10.43 for depression and 14.58 for antidepressant use, both exceeding the conventional threshold of 10. The modified Cochran’s Q test provided no statistical evidence of excess heterogeneity (Q = 61.97, *p* = 0.085), and the multivariable MR-Egger intercept provided no statistical evidence of directional horizontal pleiotropy (intercept = −0.011, SE = 0.013, *p* = 0.362). The mutually adjusted estimate was OR = 1.194 (95% CI: 0.957–1.489, *p* = 0.117) for depression and OR = 0.655 (95% CI: 0.451–0.951, *p* = 0.026) for antidepressant use. The antidepressant-use estimate provided suggestive evidence of an inverse association, although it did not meet the Bonferroni-corrected significance threshold of *p* < 0.025 (Table S5).

### 3.5. PheWAS for Pleiotropy Screening

PheWAS identified no potentially relevant secondary associations for the depression instruments within the prespecified phenotype categories. In contrast, three instrumental SNPs for antidepressant use (rs2517645, rs362331, and rs7975763) showed genome-wide significant associations with traits related to inflammation, psychiatric comorbidity, and/or socioeconomic factors. Detailed secondary associations and the corresponding IEU OpenGWAS identifiers are provided in Table S6. Exclusion of these variants yielded an IVW estimate consistent with the primary analysis (OR = 0.707, 95% CI: 0.505–0.991, *p* = 0.044).

### 3.6. Sample Overlap Analysis

Bivariate LDSC showed that the cross-trait intercepts were 0.0023 (standard error = 0.0044) for depression and tuberculosis sequelae and 0.0041 (standard error = 0.0046) for antidepressant use and tuberculosis sequelae, both of which were within one standard error of zero, indicating no or negligible overlap-related error covariance (Table S7). In the MRlap analysis, the corrected estimates remained directionally consistent with the primary IVW results (depression: corrected OR = 1.194, 95% CI: 1.004–1.419, corrected *p* = 0.045; antidepressant use: corrected OR = 0.889, 95% CI: 0.790–0.999, corrected *p* = 0.049) (Table S8).

## 4. Discussion

To the best of our knowledge, this is the first MR study to investigate the potential causal associations of genetically predicted depression and antidepressant use with the risk of tuberculosis sequelae. The primary analyses provided suggestive evidence that genetic predisposition to depression and antidepressant use may be associated with the risk of tuberculosis sequelae. Although neither association met the Bonferroni-corrected significance threshold and nominal statistical significance was not retained in the multiplicative random-effects IVW sensitivity analyses, the effect directions remained generally consistent across complementary MR analyses. Accordingly, the findings should be interpreted as exploratory and hypothesis-generating.

Previous observational studies have established that depression adversely affected tuberculosis prognosis [10,11,12,13]. For instance, depression was recognized as a significant risk factor for frailty among older adult patients with pulmonary tuberculosis in a retrospective case–control study of 204 individuals [10]. Another cross-sectional study based on community-level data from the World Health Survey (WHS) on 242,952 adults indicated that tuberculosis patients with comorbid depression had an elevated risk for declining health status scores compared to patients without depression [11]. Additionally, a prospective study of 648 adults undergoing tuberculosis treatment at 14 primary health-care facilities found that untreated depression in people with tuberculosis was associated with worse treatment outcomes, poorer quality of life, and greater disability [12]. Moreover, a systematic review identified depression as a contributor to unfavorable treatment outcomes among tuberculosis patients in India [13]. These observations highlight the clinical relevance of depression in tuberculosis populations and support further investigation of interventions addressing depression and related psychosocial factors. For example, in a 5-year retrospective study of 285 patients with multidrug-resistant tuberculosis in Peru, the addition of psychosocial therapy to standard anti-tuberculosis treatment significantly improved treatment adherence, completion, and social rehabilitation [37]. However, direct population-level evidence specifically on whether antidepressant pharmacotherapy can mitigate the risk of tuberculosis sequelae remains limited. Preclinical in vitro studies have suggested that certain antidepressants, such as fluoxetine, may exhibit additive antibacterial effects when combined with anti-tuberculosis therapy [14,15].

Although these studies offered important population-level insights into the association between depression and adverse tuberculosis clinical outcomes, the bias due to residual confounding and reverse causation could not be fully addressed due to the inherent methodological constraints of conventional observational designs. Moreover, direct epidemiological evidence regarding the impact of antidepressant use on tuberculosis sequelae has not been well established. To address these issues and to reduce the influence of confounding and reverse causation that may affect conventional epidemiological studies, we employed an MR framework. Our findings provided suggestive genetic evidence that genetically predicted depression and antidepressant use may be associated with the risk of tuberculosis sequelae. The multivariable MR analysis yielded estimates that were directionally consistent with the univariable analyses; however, given the relaxed instrument-selection threshold, these findings should also be interpreted cautiously. Overall, the findings should be regarded as exploratory and hypothesis-generating rather than confirmatory evidence of causal effects. In particular, the antidepressant-use GWAS reflects genetic liability to a broad medication-use phenotype rather than the pharmacological effects of antidepressant treatment.

The opposing associations observed in our study may be explained by psychosocial and biological mechanisms. Psychosocially, depression compromises key health behaviors such as medication adherence and treatment completion, which are critical drivers of tuberculosis sequelae risk [12,38]. Biologically, depression is characterized by a chronic pro-inflammatory state and neuroendocrine dysregulation, which could impair the cell-mediated immunity against Mtb and hinder post-infection tissue repair, thereby increasing the risk of tuberculosis sequelae [39,40]. The mechanisms underlying the inverse association with genetically predicted antidepressant use remain uncertain. Although previous studies have suggested that improvement of depressive symptoms may enhance medication adherence and treatment completion [8,41], and that certain antidepressants such as fluoxetine may exert immunomodulatory or antimicrobial effects against Mtb [15], these mechanisms cannot be directly inferred from the present MR analysis. This is because the antidepressant-use GWAS reflects genetic liability to a broad medication-use phenotype rather than exposure to a specific pharmacological treatment. Further studies are needed to clarify the biological and behavioral pathways underlying this association.

Our findings may have potential clinical and public health relevance. Tuberculosis sequelae represent a major source of long-term disability and healthcare burden worldwide [7], and depression is a prevalent yet frequently under-addressed comorbidity in tuberculosis patients [9,11,42]. This MR study provided suggestive genetic evidence that genetically predicted depression and antidepressant use may be associated with the risk of tuberculosis sequelae. However, these findings are exploratory and hypothesis-generating and do not establish that depression treatment or antidepressant pharmacotherapy can prevent tuberculosis sequelae. In public health terms, the observed associations highlight the need for further investigation of the relationship between mental health and long-term tuberculosis outcomes. We hypothesized that patients with tuberculosis and comorbid depression, especially if untreated, may represent a subgroup warranting further investigation with respect to long-term sequelae risk. Further clinical studies are warranted to determine the clinical relevance of these associations, including whether depression management or antidepressant treatment influences long-term outcomes in tuberculosis populations.

This study has several strengths. First, as far as we know, this is the first MR study assessing the potential causal roles of depression and antidepressant use in the risk of tuberculosis sequelae. Taking advantage of the random allocation of genetic variants at conception, the MR framework may reduce, although not eliminate, the influence of confounding and reverse causation compared with conventional observational analyses. Second, our analysis was based on large-scale GWAS summary statistics, which provided a substantial sample size for the MR analyses. Third, the primary findings showed generally consistent directions across a suite of sensitivity analyses, and the MR-PRESSO global test and MR-Egger intercept provided no statistical evidence of horizontal pleiotropy, although residual pleiotropy cannot be excluded.

However, some limitations should also be discussed here. First, the GWAS summary statistics used were derived from European-ancestry populations, which limited the generalizability of our findings to other ethnic groups. Second, the depression exposure was based on clinically coded depression phenotypes in UK Biobank and FinnGen and may not capture the full spectrum of depressive disorders; therefore, the findings should not be generalized to all forms of depression. In addition, the genetic instruments for antidepressant use were based on a broad phenotype of “any antidepressant use”, and thus could not distinguish between antidepressant initiation, specific drug classes, dosages, treatment durations, adherence, or treatment timing. Moreover, genetic liability to antidepressant use may capture underlying depression liability, disease severity, healthcare-seeking behavior, prescribing patterns, other indications for antidepressant use, and related comorbidities rather than the pharmacological effects of antidepressants per se; therefore, the observed inverse association should not be interpreted as evidence that antidepressant treatment prevents tuberculosis sequelae. Third, MR estimates reflected the lifelong effect of genetic predisposition to depression and antidepressant use, which may differ from the effect of initiating treatment in a clinical setting. Fourth, the FinnGen tuberculosis sequelae phenotype was based on the broad ICD-10 B90 category and therefore encompassed clinically heterogeneous sequelae involving different organ systems. As this endpoint was derived from registry-based diagnostic codes, some degree of phenotype misclassification cannot be excluded. Moreover, according to the FinnGen control definition, individuals with a history of tuberculosis but without a recorded B90 sequela diagnosis could have been included in the control group. Because subtype-specific GWAS summary statistics, including those for pulmonary sequelae, were not publicly available, separate MR analyses for individual sequelae could not be performed. Fifth, the relatively liberal SNP selection threshold (*p* < 5 × 10^−6^) may increase susceptibility to weak-instrument bias, false-positive instruments, winner’s curse, and pleiotropy. Although all selected instruments had F-statistics >10 and no statistical evidence of horizontal pleiotropy was detected by MR-PRESSO or MR-Egger, residual pleiotropy cannot be fully excluded. Moreover, application of the conventional genome-wide significance threshold (*p* < 5 × 10^−8^) yielded no eligible instruments for depression and only one for antidepressant use, precluding a meaningful multi-variant MR sensitivity analysis at this threshold. Accordingly, the findings based on the relaxed instrument-selection threshold should be interpreted with caution. In addition, the study had limited statistical power to detect an antidepressant-use effect of the observed magnitude; at 80% power and a two-sided α level of 0.05, the minimum detectable OR for an inverse association was 0.623, whereas the observed primary OR was 0.692. Sixth, although the MRlap-corrected estimates remained directionally consistent with the primary IVW results, the antidepressant-use estimate was attenuated from OR = 0.692 to OR = 0.889. Because MRlap simultaneously accounts for sample overlap, weak-instrument bias, and winner’s curse, the source of this attenuation cannot be attributed to any single factor. Moreover, although no cohort-level overlap was expected between the UK Biobank antidepressant-use GWAS and the FinnGen outcome dataset, the exact number and proportion of overlapping participants could not be determined from the available summary-level data. Therefore, the attenuation after MRlap correction suggests that the magnitude of the primary antidepressant-use estimate should be interpreted cautiously. Finally, the current lack of independent GWAS datasets with comparable definitions of tuberculosis sequelae and publicly available summary statistics limited our ability to perform independent replication of the findings. Future studies should aim to validate these associations in more diverse populations, investigate the effects of specific antidepressant classes and dosages, evaluate associations across more granular tuberculosis sequelae phenotypes, particularly pulmonary sequelae, and replicate the present findings using a larger number of genome-wide significant genetic instruments as larger GWAS datasets become available. Further prospective studies are also needed to clarify the clinical relevance of these associations and to determine whether they extend to clinically defined depression and antidepressant treatment exposures.

## 5. Conclusions

In conclusion, this exploratory MR study provided novel, hypothesis-generating genetic evidence that genetically predicted depression and antidepressant use may be associated with the risk of tuberculosis sequelae. These findings highlight potentially important links between mental health-related factors and long-term tuberculosis outcomes, but should not be interpreted as evidence that depression treatment or antidepressant pharmacotherapy prevents tuberculosis sequelae. Further studies are warranted to validate these associations in independent datasets, elucidate the underlying biological and behavioral mechanisms, and clarify their clinical relevance through well-designed pharmacoepidemiological studies and clinical trials.

## Figures and Tables

**Figure 1:**
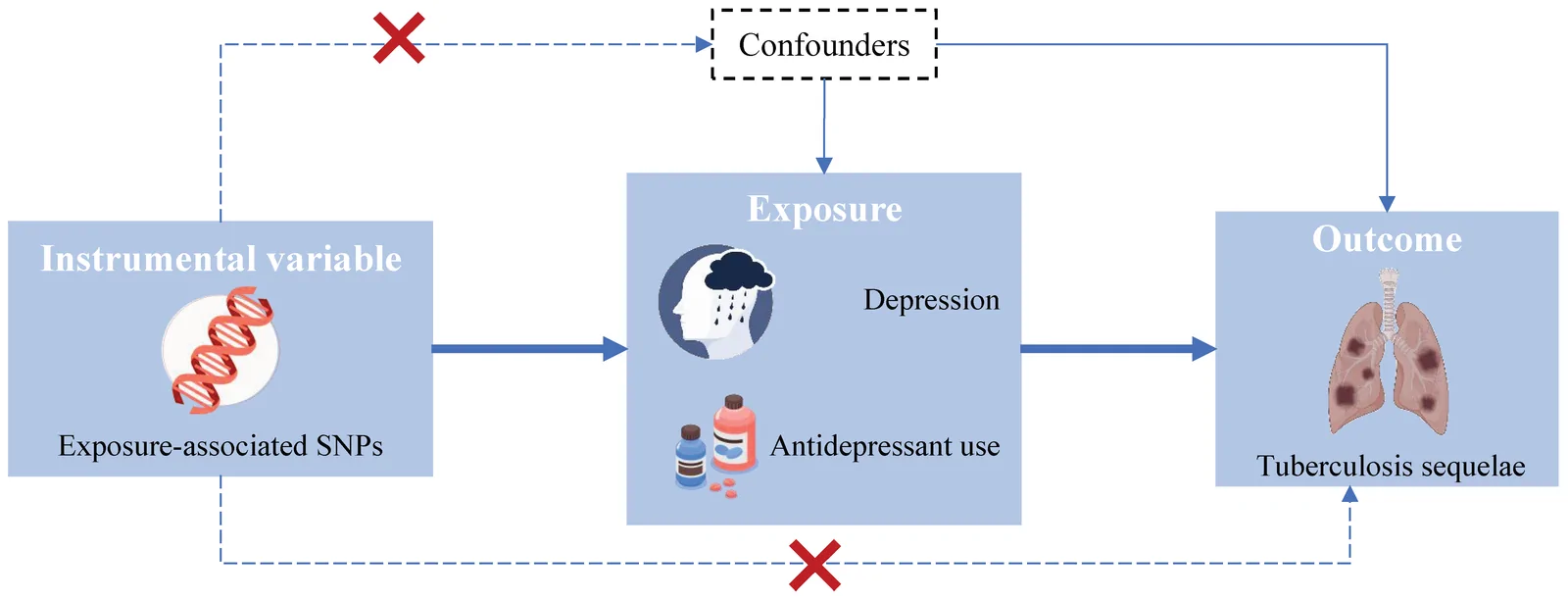
Conceptual framework for Mendelian randomization analysis of the association of depression and antidepressant use with the risk of tuberculosis sequelae. There are three core assumptions for Mendelian randomization design: (1) instrumental variables are strongly associated with the exposure; (2) instrumental variables are not associated with any confounders related to exposure and outcome; and (3) instrumental variables affect outcomes only through the effects on exposures. Abbreviation: SNP, single-nucleotide polymorphism.

**Figure 2:**
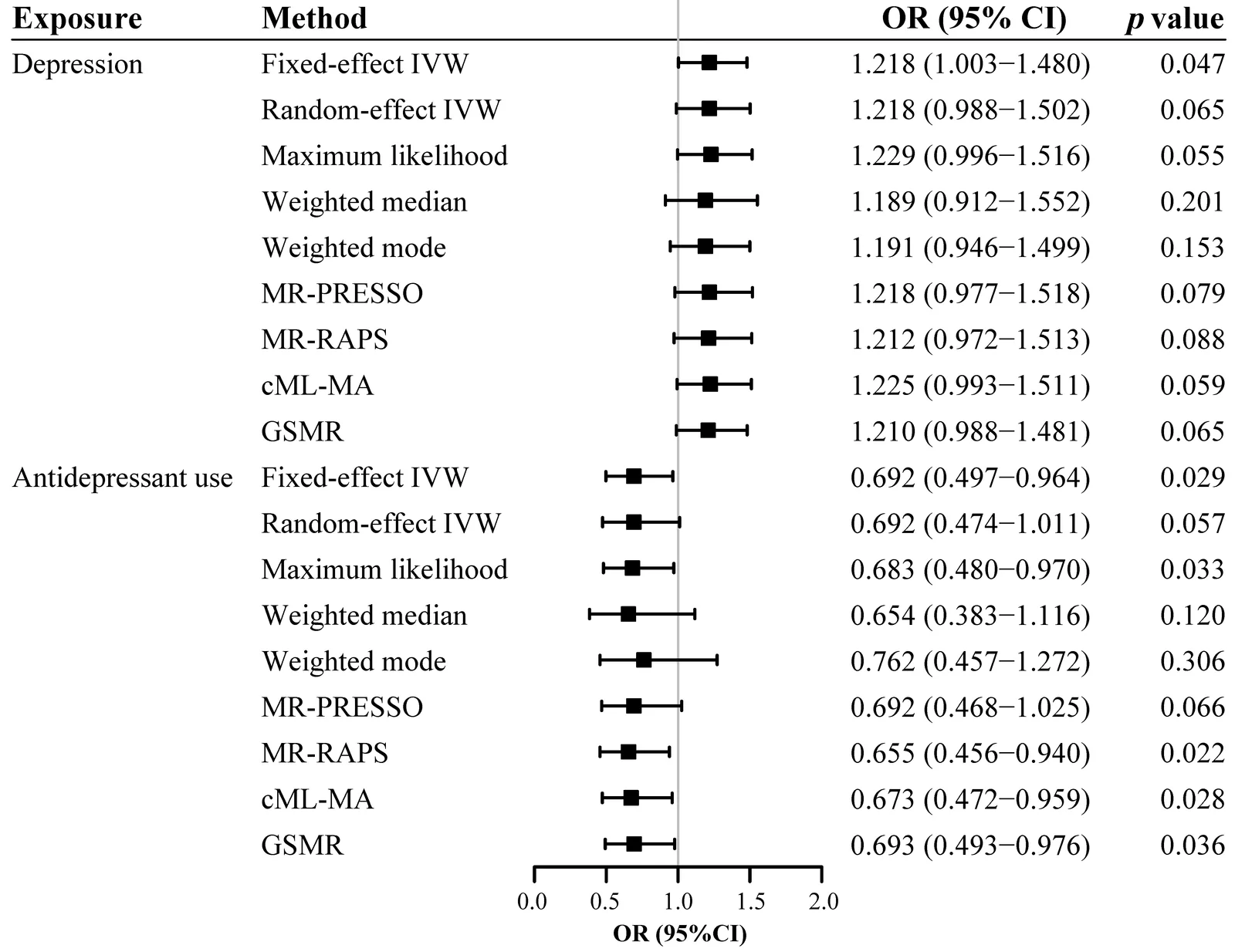
Summary Mendelian randomization estimates for the associations of depression and antidepressant use with the risk of tuberculosis sequelae. Abbreviations: CI, confidence interval; cML-MA, constrained maximum likelihood and model averaging; GSMR, generalized summary data-based Mendelian randomization; IVW, inverse-variance weighted; MR-PRESSO, Mendelian randomization pleiotropy residual sum and outlier; MR-RAPS, Mendelian randomization robust adjusted profile score; OR, odds ratio.

**Figure 3:**
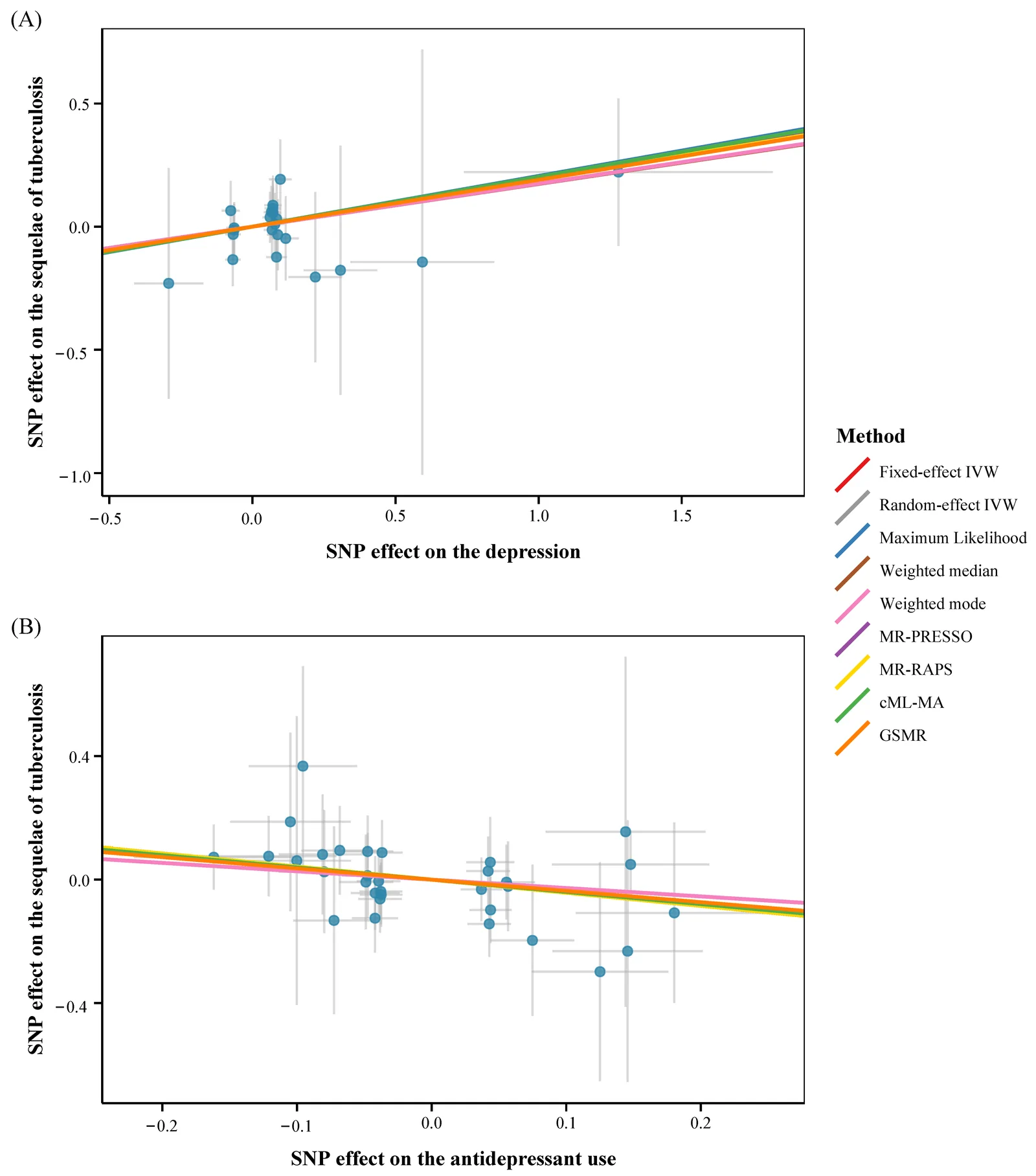
Scatterplots for the associations of depression and antidepressant use with the risk of tuberculosis sequelae: (**A**) depression; (**B**) antidepressant use. The slope of the line indicates the estimated effect using the corresponding Mendelian randomization method. Circles indicate marginal genetic associations with exposures and risk of outcome for each variant. Horizontal and vertical lines through each circle represent the corresponding 95% confidence intervals for the genetic associations. Abbreviations: cML-MA, constrained maximum likelihood and model averaging; GSMR, generalized summary data-based Mendelian randomization; IVW, inverse-variance weighted; MR-PRESSO, Mendelian randomization pleiotropy residual sum and outlier; MR-RAPS, Mendelian randomization robust adjusted profile score; SNP, single-nucleotide polymorphism.

**Table 1:** Detailed information on data sources.

Phenotype	Consortium	Publicly Available Year	Sample Size (Case/Control)	Population	URL	PMID
**Exposure**						
Depression	Biobank + FinnGen (release 3)	2021	449,414 (13,559/435,855)	European	https://pheweb.jp/downloads (accessed on 28 February 2026)	34,594,039
Antidepressant use	Biobank	2021	304,162 (33,757/270,405)	European	https://pheweb.jp/downloads (accessed on 28 February 2026)	34,594,039
**Outcome**						
Tuberculosis sequelae	FinnGen (release 12)	2024	497,887 (727/497,160)	European	https://storage.googleapis.com/finngen-public-datar12/summary_stats/release/finngen_R12_AB1_SEQULAE_TUBERCU.gz (accessed on 28 February 2026)	36,653,562

Abbreviation: PMID, PubMed Unique Identifier; URL, Uniform Resource Locator.

**Table 2:** Pleiotropy assessment for the associations of depression and antidepressant use with the risk of tuberculosis sequelae.

Exposure	MR-PRESSO Global Test		MR-Egger Intercept	
	Observed RSS	*p* Value	Beta (SE)	*p* Value
Depression	25.401	0.418	0.019 (0.019)	0.331
Antidepressant use	43.906	0.129	0.006 (0.027)	0.821

Abbreviations: MR-PRESSO, Mendelian randomization pleiotropy residual sum and outlier; RSS, Residual Sum of Squares; SE, standard error.

## Data Availability

All summary-level data used in the present study are available online from the corresponding GWASs via the URL provided in Table 1. The main analysis code used in this study is available at https://github.com/Yana728/MRcode/tree/Dep/ATD-TB (accessed on 10 September 2026).

## Supplementary Materials

The following supporting information can be downloaded at: https://www.mdpi.com/article/10.3390/genes17101198/s1, Table S1. Characteristics of genetic variants as instrumental variables for depression; Table S2. Characteristics of genetic variants as instrumental variables for antidepressant use; Table S3. Results of heterogeneity test and inverse-variance-weighted model used in the analysis; Table S4. The results of the Mendelian randomization Steiger test; Table S5. Multivariable Mendelian randomization estimates for the associations of depression and antidepressant use with the risk of tuberculosis sequelae; Table S6. Secondary associations of instrumental variants identified by phenome-wide screening; Table S7. Cross-trait LDSC assessing potential sample overlap between exposure and outcome GWASs; Table S8. MRlap sensitivity analysis for the associations of depression and antidepressant use with the risk of tuberculosis sequelae; Figure S1. Flow diagram of instrumental variant selection and harmonization for depression and antidepressant use; Figure S2. Leave-one-out analyses for the associations of depression and antidepressant use with the risk of tuberculosis sequelae; Figure S3. Funnel plots for the associations of genetically predicted depression and antidepressant use with the risk of tuberculosis sequelae.
