# Supplementary material for: Associations of Depression and Antidepressant Use with the Risk of Tuberculosis Sequelae: Insight from Genetic Analysis
Source: Genes (Basel). 2026 Sep 28;17(10):1198. doi: 10.3390/genes17101198 (PMC13704685; doi:10.3390/genes17101198)
Supplement: Supplementary file 1 [file genes-17-01198-s001.zip › genes-4525032-supplementary.pdf]

Supplementary materials

Table S1. Characteristics of genetic variants as instrumental variables for depression.

Table S2. Characteristics of genetic variants as instrumental variables for antidepressant use.

Table S3. Results of heterogeneity test and inverse-variance-weighted model used in the analysis.

Table S4. The results of the Mendelian randomization Steiger test.

Table S5. Multivariable Mendelian randomization estimates for the associations of depression and antidepressant use with the risk of tuberculosis sequelae.

Table S6. Secondary associations of instrumental variants identified by phenome-wide screening.

Table S7. Cross-trait LDSC assessing potential sample overlap between exposure and outcome GWASs.

Table S8. MRlap sensitivity analysis for the associations of depression and antidepressant use with the risk of tuberculosis sequelae.

Figure S1. Flow diagram of instrumental variant selection and harmonization for depression and antidepressant use.

Figure S2. Leave-one-out analyses for the associations of depression and antidepressant use with the risk of tuberculosis sequelae.

Figure S3. Funnel plots for the associations of genetically predicted depression and antidepressant use with the risk of tuberculosis sequelae.

Table S1. Characteristics of genetic variants as instrumental variables for depression.

Chr	Position (GRCh37)	SNP	EA	OA	Beta	SE	*p* value	F-statistic
1	38569012	rs34608209	T	G	0.5941	0.1282	3.61E-06	21.48
1	98833455	rs10493901	G	T	-0.2924	0.0617	2.18E-06	22.46
1	111629006	rs111364974	T	C	1.2790	0.2751	3.33E-06	21.62
1	226545866	rs6668851	A	C	-0.0682	0.0140	1.03E-06	23.73
2	38516428	rs13417898	C	T	-0.0671	0.0139	1.39E-06	23.30
2	208329196	rs35130355	T	C	-0.0635	0.0137	3.49E-06	21.48
3	43486923	rs1842806	T	C	-0.0639	0.0139	4.63E-06	21.13
3	191887975	rs2046918	A	G	0.0717	0.0155	3.88E-06	21.40
5	11816618	rs116127119	A	G	0.3078	0.0653	2.44E-06	22.22
6	125057899	rs1856246	G	T	0.0619	0.0135	4.73E-06	21.02
6	149884597	rs9498371	C	T	0.0666	0.0138	1.43E-06	23.29
6	155887039	rs189504242	C	A	0.2200	0.0478	4.09E-06	21.18
8	65960510	rs10957334	A	G	0.0715	0.0144	7.30E-07	24.65
8	78362277	rs7835373	A	G	0.0846	0.0184	4.21E-06	21.14
10	80838651	rs7920460	T	C	0.0978	0.0202	1.29E-06	23.44
10	126156923	rs4962579	T	C	0.0723	0.0140	2.47E-07	26.67
16	4817433	rs74702231	T	A	0.0787	0.0169	3.07E-06	21.69
16	26830227	rs17795658	C	T	0.0887	0.0191	3.52E-06	21.57
16	73140324	rs8061978	T	C	0.0692	0.0151	4.34E-06	21.00
18	40856982	rs73487503	T	C	0.1164	0.0233	5.72E-07	24.96
19	631574	rs145176510	A	G	0.0843	0.0183	3.90E-06	21.22
22	49700618	rs916241	C	T	-0.0755	0.0162	3.15E-06	21.72

Abbreviations: Chr, chromosome; EA, effect allele; OA, other allele; SE, standard error; SNP, single nucleotide polymorphism.

Table S2. Characteristics of genetic variants as instrumental variables for antidepressant use.

Chr	Position (GRCh37)	SNP	EA	OA	Beta	SE	*p* value	F-statistic
1	118887437	rs17460214	C	G	-0.0682	0.0145	2.49E-06	22.12
2	15843584	rs7566672	T	A	-0.0419	0.0092	4.93E-06	20.74
2	104535716	rs1441104	A	G	-0.0377	0.0081	3.64E-06	21.66
4	3215835	rs362331	C	T	-0.0368	0.0078	2.54E-06	22.26
5	38007419	rs10941376	C	G	0.1478	0.0298	7.07E-07	24.60
5	87826775	rs78640982	T	A	0.0568	0.0123	4.02E-06	21.32
5	105775757	rs114373539	C	A	0.1803	0.0373	1.33E-06	23.37
6	30122623	rs2517645	C	T	-0.0488	0.0103	2.09E-06	22.45
6	105397418	rs395962	G	T	0.0420	0.0083	4.32E-07	25.61
6	133787110	rs212767	G	T	-0.1618	0.0302	8.17E-08	28.70
7	12253362	rs12699323	C	T	0.0428	0.0082	1.84E-07	27.24
7	89376214	rs2188685	A	G	0.1456	0.0285	3.31E-07	26.10
7	100985415	rs150214947	G	T	-0.0956	0.0205	3.23E-06	21.75
7	114056460	rs12705964	G	A	-0.0810	0.0166	1.09E-06	23.81
7	156112739	rs114269832	G	A	-0.1210	0.0257	2.51E-06	22.17
8	127338287	rs7834144	T	A	-0.0420	0.0087	1.54E-06	23.31
9	37106370	rs62535670	A	T	0.0556	0.0108	2.46E-07	26.50
10	12812504	rs12784393	G	A	-0.0393	0.0083	2.40E-06	22.42
12	1820987	rs35666763	A	T	-0.0725	0.0154	2.56E-06	22.16
12	16310816	rs10846305	G	A	0.0438	0.0080	4.24E-08	29.98
12	101015056	rs75215063	G	A	0.1441	0.0303	1.93E-06	22.62
12	123001692	rs78525750	G	A	0.0749	0.0158	2.21E-06	22.47
12	123604053	rs7975763	T	C	-0.0475	0.0097	1.07E-06	23.98
13	21233538	rs9509322	C	T	-0.0475	0.0097	8.56E-07	23.98
13	86498660	rs137946645	G	T	-0.1001	0.0205	1.00E-06	23.84
13	100592264	rs7317261	T	C	-0.0374	0.0081	3.81E-06	21.32
14	62374092	rs2023211	C	T	0.0436	0.0091	1.57E-06	22.96
17	74206117	rs12942176	G	A	0.0370	0.0079	2.73E-06	21.94
18	23841849	rs62085367	A	G	-0.1049	0.0228	4.31E-06	21.17
19	3285042	rs113646375	T	C	-0.0383	0.0083	4.14E-06	21.29
20	1065987	rs80275385	A	G	-0.0797	0.0173	4.22E-06	21.22
20	57441972	rs77057913	G	C	0.1251	0.0259	1.37E-06	23.33

Abbreviations: Chr, chromosome; EA, effect allele; OA, other allele; SE, standard error; SNP, single nucleotide polymorphism.

Table S3. Results of heterogeneity test and inverse-variance-weighted model used in the analysis.

Exposure	Q value	*p* value	Model
Depression	24.30	0.279	fixed effect model
Antidepressant use	40.57	0.117	fixed effect model

Table S4. The results of the Mendelian randomization Steiger test.

Exposure	R^2 for exposure	R^2 for outcome	Correct causal direction	$p_{Steiger}$
Depression	1.10×10^{-3}	5.67E-05	TRUE	1.78×10^{-35}
Antidepressant use	2.46×10^{-3}	9.10E-05	TRUE	5.99×10^{-68}

Table S5. Multivariable Mendelian randomization estimates for the associations of depression and antidepressant use with the risk of tuberculosis sequelae.

Exposure	OR (95%CI)	*p* value	F-statistic
Depression	1.194 (0.957-1.489)	0.117	9.43
Antidepressant use	0.655 (0.451-0.951)	0.026	14.58

Abbreviations: CI, confidence interval; OR, odds ratio.

Table S6. Secondary associations of instrumental variants identified by phenome-wide screening.

Exposure	SNP	Secondary phenotype	Category	IEU OpenGWAS ID	PMID	*p* value
Antidepressant use	rs2517645	White blood cell count	Inflammation	ieu-b-30	32888493	6.30×10^{-137}
Antidepressant use	rs2517645	Schizophrenia	Psychiatric comorbidity	ieu-b-5102	35396580	1.97×10^{-20}
Antidepressant use	rs362331	White blood cell count	Inflammation	ieu-b-30	32888493	3.38×10^{-12}
Antidepressant use	rs362331	Educational attainment (college completion)	Socioeconomic factor	ebi-a-GCST90029012	29892013	3.40×10^{-10}
Antidepressant use	rs7975763	Lymphocyte count	Inflammation	ieu-b-32	32888493	1.37×10^{-23}
Antidepressant use	rs7975763	Educational attainment (college completion)	Socioeconomic factor	ebi-a-GCST90029012	29892013	1.70×10^{-9}
Antidepressant use	rs7975763	Schizophrenia	Psychiatric comorbidity	ieu-b-5102	35396580	1.43×10^{-9}

Abbreviations: GWAS, genome-wide association study; PMID, PubMed Unique Identifier; SNP, single nucleotide polymorphism.

Table S7. Cross-trait LDSC assessing potential sample overlap between exposure and outcome GWASs.

Exposure	Cross-trait LDSC intercept	SE of intercept
Depression	0.0023	0.0044
Antidepressant use	0.0041	0.0046

Abbreviations: GWASs, genome-wide association studies; LDSC, linkage disequilibrium score regression; SE, standard error.

The cross-trait LDSC intercept reflects error covariance between GWAS summary statistics that may arise from sample overlap and other shared sources of bias. An intercept close to zero indicates little evidence of substantial cross-trait error covariance. The intercept does not directly quantify the number or proportion of overlapping participants.

Table S8. MRlap sensitivity analysis for the associations of depression and antidepressant use with the risk of tuberculosis sequelae.

Exposure	Corrected OR (95%CI)	Corrected *p*	*p* for difference
Depression	1.194 (1.004-1.419)	0.045	0.053
Antidepressant use	0.889 (0.790-0.999)	0.049	0.042

Abbreviations: CI, confidence interval; OR, odds ratio.

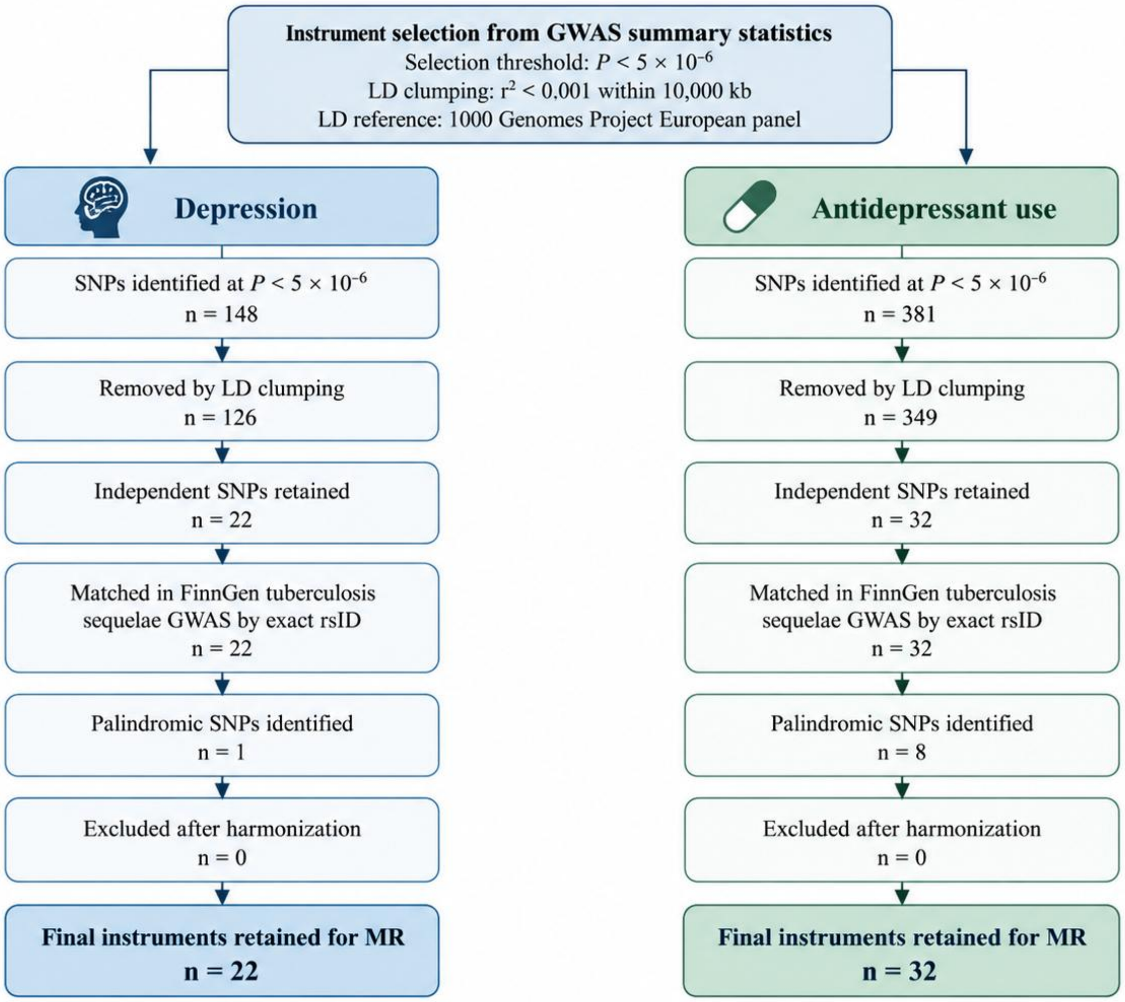

Figure S1. Flow diagram of instrumental variant selection and harmonization for depression and antidepressant use.

Abbreviations: GWAS, genome-wide association study; LD, linkage disequilibrium; MR, Mendelian randomization; SNPs, single nucleotide polymorphisms.

SNP selection was performed using the extract_instruments function in TwoSampleMR. LD clumping was based on the European reference panel of the 1000 Genomes Project. Harmonization was performed using harmonise_data with action = 2. No proxy SNPs were used because all clumped variants were directly available in the outcome dataset.

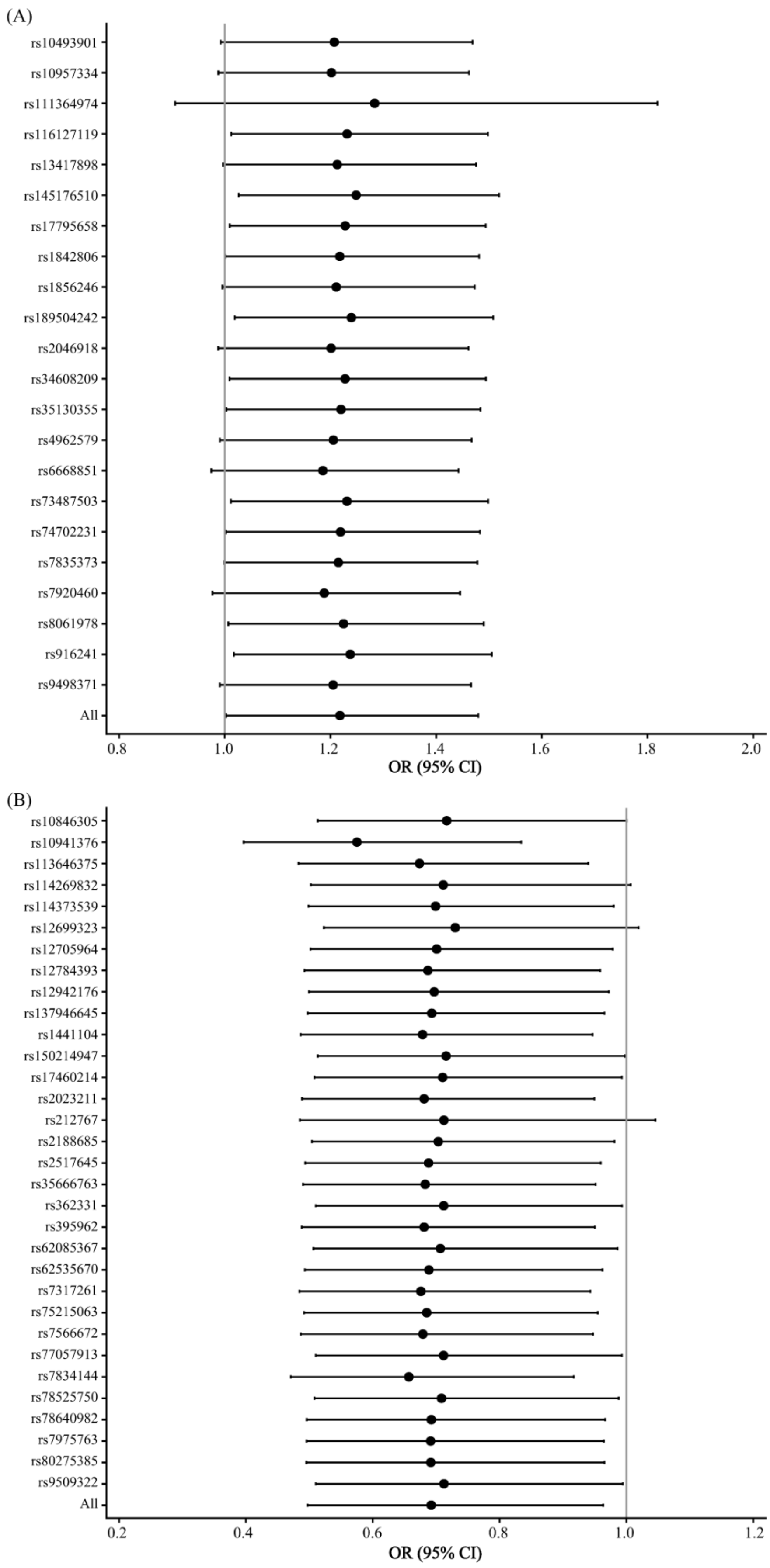

Figure S2. Leave-one-out analyses for the associations of depression and antidepressant use with the risk of tuberculosis sequelae. (A) depression; (B) antidepressant use.

Abbreviations: CI, confidence interval; OR, odds ratio.

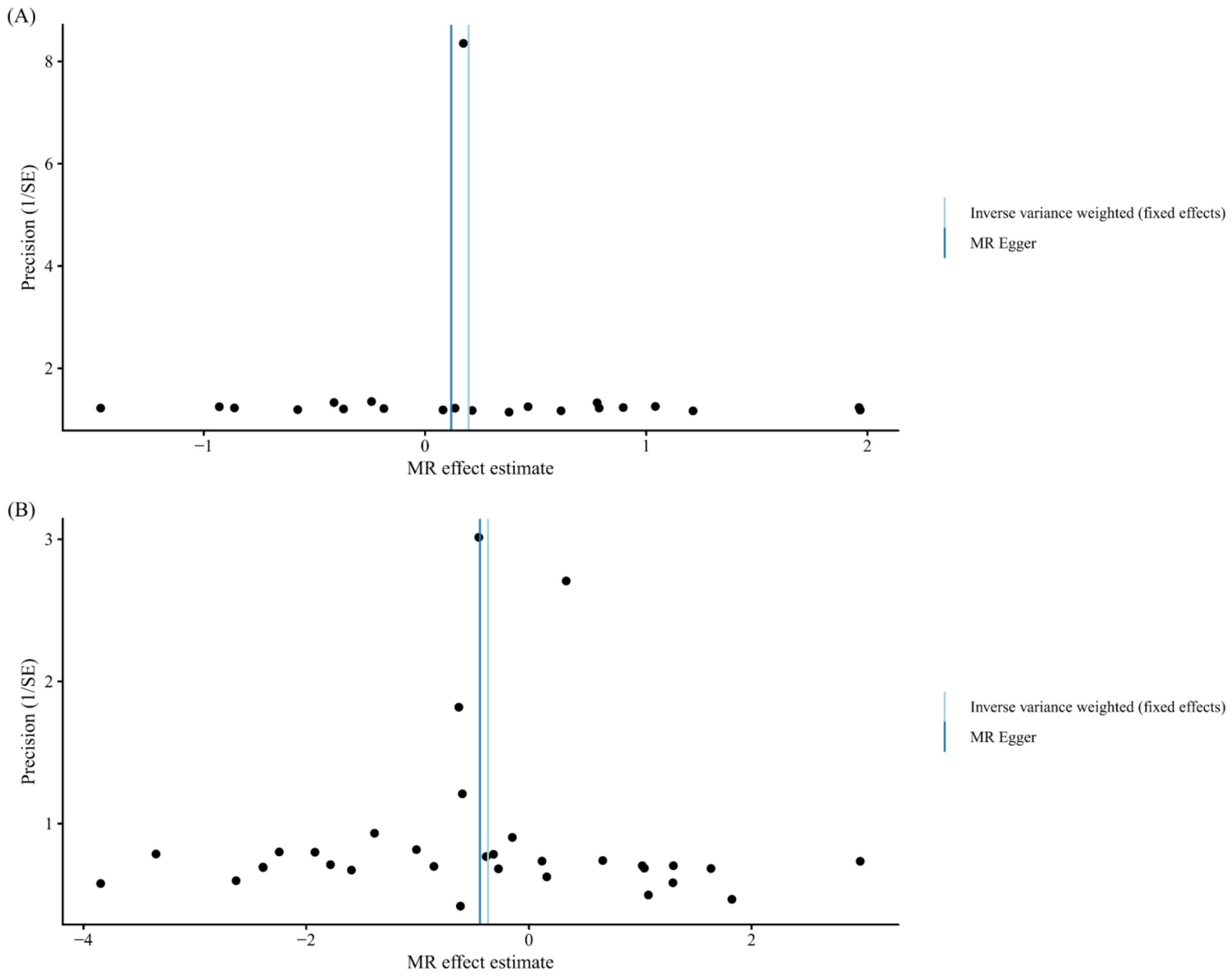

Figure S3. Funnel plots for the associations of genetically predicted depression and antidepressant use with the risk of tuberculosis sequelae. (A) depression; (B) antidepressant use.

Abbreviations: MR, Mendelian randomization.
